# Maternal Body Mass Index, Myometrium Contractility and Uterotonic Receptor Expression in Pregnancy

**DOI:** 10.1007/s43032-024-01661-1

**Published:** 2024-07-25

**Authors:** Sydney M. Lammers, Kyra K. Peczkowski, Niharika Patel, Mahmoud Abdelwahab, Taryn L. Summerfield, Maged M. Costantine, Paul M. L. Janssen, Douglas A. Kniss, Heather A. Frey

**Affiliations:** 1 Department of Obstetrics and Gynecology, Division of Maternal-Fetal Medicine, The Ohio State University College of Medicine, 395 W. 12Th Ave, 5Th Floor, Columbus, OH USA; 2 Department of Physiology and Cell Biology, The Ohio State University, College of Medicine, Columbus, OH USA; 3 Laboratory of Perinatal Research, The Ohio State University, Wexner Medical Center, Columbus, OH USA

**Keywords:** Contractility, Labor dysfunction, Myometrium, Obesity, Oxytocin receptor

## Abstract

Pregnant individuals with obesity (body mass index, BMI ≥ 30 kg/m^2^) are more likely to experience prolonged labor and have double the risk of cesarean compared with individuals with normal weight (BMI < 25 kg/m^2^). The aim of this study was to evaluate whether obesity in pregnancy is associated with reduced spontaneous and oxytocin-stimulated myometrial contractile activity using ex vivo preparations. We also assessed the relationship between maternal BMI and the expression of oxytocin (OXTR) and prostaglandin (FP) receptors in the myometrial tissue. We enrolled 73 individuals with a singleton gestation undergoing scheduled cesarean delivery at term in a prospective cohort study. This included 49 individuals with a pre-pregnancy BMI ≥ 30 kg/m^2^ and 24 with BMI < 25.0 kg/m^2^. After delivery, a small strip of myometrium was excised from the upper edge of the hysterotomy. Baseline spontaneous and oxytocin stimulated myometrial contractile activity was measured using ex vivo preparations. Additionally, expression of oxytocin and prostaglandin receptors from myometrial samples were compared using qRT-PCR and western blot techniques. Spontaneous and oxytocin-stimulated contraction frequency, duration, and force were not significantly different in myometrial samples from the obese and normal-weight individuals. Myometrial OXTR gene and protein expression was also similar in the two groups. While FP gene expression was lower in the myometrial samples from the obese group, protein expression did not differ. These data help to address an important knowledge gap related to the biological mechanisms underlying the association between maternal obesity and dysfunctional labor.

## Introduction

Obesity (body mass index, BMI $\ge$ 30 kg/m^2^) is a serious and growing public health problem. In the United States, it is estimated that almost one third of pregnant individuals have obesity [1]. Pregnant individuals with obesity experience higher rates of obstetric complications, such as hypertensive disorders [2, 3] and gestational diabetes mellitus, compared to individuals with normal weight (BMI 18.5–24.9 kg/m^2^) [4, 5]. In addition, obesity is a risk factor for fetal macrosomia and adverse neonatal outcomes [5–7].

Individuals with obesity are also are more than twice as likely to deliver by cesarean compared with those of normal-weight, with cesarean rates as high as 50% in individuals with class III obesity (BMI $\ge$ 40 kg/m^2^) [8–10]. In the United States, labor arrest is a leading indication for cesarean in all pregnant individuals, but a significantly higher proportion of cesarean deliveries in individuals with obesity are for this indication than for those with lower BMI [9]. Several studies evaluating labor curves have shown that increasing maternal BMI is associated with slower progression of cervical dilation in labor [11, 12]. Moreover, obesity increases the risk for post-operative complications among those who deliver by cesarean, including postpartum hemorrhage and infection [13–15]. The relationship between obesity in pregnancy and dysfunctional labor is well-established, however, a biological explanation has yet to be defined.

A leading hypothesis is that obesity directly affects intrinsic contractile properties within the myometrium, but the findings in prior studies evaluating contractility have been conflicting. In one of the first studies comparing in vitro myometrial contractility and maternal BMI, Zhang et al. [16] found that myometrial samples from individuals with obesity contracted less frequently and with less force than myometrial samples from those with normal weight. Follow up studies reported inconsistent association between obesity and contractile measures of myometrial tissue, such as frequency and time to onset of contractions [17, 18], while other investigators have failed to demonstrate an effect of obesity on myometrial contractile responses to oxytocin [19].

Further research on the biomolecular mechanisms by which obesity affects myometrial function is also needed. The neuropeptide oxytocin stimulates myometrial contractions through multiple pathways [20], but there is a paucity of data on the association between maternal BMI and oxytocin signaling. In a study of 20 individuals, Garbedian et al. found a positive correlation between myometrial expression of oxytocin receptor (OXTR) and maternal BMI [21], but in a slightly larger study Grotegut et al. [22] reported that neither *OXTR* gene or OXTR protein expression was affected by maternal BMI.

Therefore, we conducted this prospective cohort study to evaluate whether obesity in pregnancy is associated with reduced spontaneous and oxytocin-stimulated myometrial contractile activity using *ex vivo* preparations. We also examined the relationship between maternal obesity status and expression of oxytocin and prostaglandin F_2α_ receptors (FP) in order to better understand differences in myometrial contractility at the molecular level. We hypothesized that myometrial tissue from term individuals with obesity have reduced expression of the OXTR and FP and decreased contractility compared with samples from normal-weight individuals.

## Methods

### Participant Enrollment

Pregnant individuals presenting to the labor and delivery unit at The Ohio State University Wexner Medical Center from March 2020 through March 2022 for scheduled cesarean delivery at term (≥ 37 weeks’ gestation) were approached for enrollment in this prospective study. Only individuals between the ages of 18–48 years with a singleton gestation and a pre-pregnancy BMI < 25.0 kg/m2 (normal BMI group) or pre-pregnancy BMI ≥ 30 kg/m2 were eligible. Individuals in labor at the time of cesarean delivery, including those with rupture of membranes and/or regular uterine contractions with cervical change were excluded. Patients with pre-existing (Type 1 or Type 2) diabetes mellitus were excluded to avoid potential metabolic confounding factors in muscle contractility experiments. Other exclusion criteria included oxytocin or prostaglandin use prior to cesarean delivery, magnesium sulfate exposure within 12 h prior to delivery, HIV, hepatitis B or hepatitis C virus or other sexually transmitted infections, suspected or confirmed chorioamnionitis, and abnormal placentation. Approval was obtained from the Ohio State University Biomedical Institutional Review Board prior to starting the study. All participants provided written informed consent.

Trained research staff collected clinical information from participants’ medical records following enrollment. This included information about maternal age, parity, race and ethnicity, gestational age, indication for cesarean and infant birthweight.

### Myometrial Tissue Collection and Processing

Myometrial tissue samples measuring approximately 3.5 cm in length and 2 cm in height were excised from the upper edge of the hysterotomy incision immediately following delivery of the fetus and placenta. Specimens were processed within 12 h of collection. The specimens were stored at 4ºC in isotonic saline containing glucose and pyruvate until processing was completed. After dissecting decidua and serosa from the specimen, the myometrial tissue was rinsed with cold physiological saline solution (PSS, 154 mM NaCl, 5.6 mM KCl, 1.2 mM MgSO_4_, 7.8 mM glucose, 2 mM CaCl_2_, and 10.9 mM 2-[4-(2-hydroxyethyl)piperazin-1-1yl]ethanesulfonic acid, HEPES, pH 7.4) and cut into four to five ~ 2 × 8 × 1 mm strips to be used for contractility experiments. The muscle strips were placed in fresh, cold PSS for transport and storage. The remaining myometrial tissue was frozen immediately and stored at -80 °C for qRT-PCR and western blot analysis.

### Reagents

Oxytocin was purchased from Tocris/BioTechne (Minneapolis, MN). Human recombinant interleukin-1β was purchased from R&D Systems (Minneapolis, MN). Protein assay reagent and bovine serum albumin (BSA) as standard, Precision Plus™ protein molecular weight standards, and Clarity™ chemiluminescent reagent were obtained from Biorad (Hercules, CA). All other chemicals were reagent grade and were purchased from Sigma/Aldrich (St. Louis, MO).

### Myometrial Contractility Studies

Contractility experiments were completed within 16 h of cesarean delivery using the technique described by Arrowsmith et al. [23]. A custom-made apparatus designed to measure contractile force of muscle tissue was used for these [24]. Fresh PSS was added to the 50 mL volume solution reservoir of the set-up and allowed to equilibrate to 37 °C (heated by a circulating water bath). The solution pump was set to allow the PSS to flow through the set-up at a slow and steady rate. A preparation bath volume of 400 µl was continuously circulated at approximately 10 refreshments per minute. Myometrium strips were mounted in the apparatus between two hooks, with one of the hooks attached to a force transducer (KG7, World Precision Instruments, Sarasota, Florida). The strips were trimmed, if necessary, to be no more than 5 mm in length in the pre-stretched state. The initial resting tension was adjusted to 0 mN/mm^2^. The myometrium was slowly stretched to optimal length with a micromanipulator until the resting tension was between 1.5–2.0 mN/mm^2^. Once optimal length and tension were achieved, the length, width, and thickness were recorded in custom-written LabView™ software (National Instruments, Austin, TX). The muscles were allowed to stabilize and begin spontaneously contracting approximately 1.5–2 h after mounting. After the first contraction, the specimens were allowed to stabilize for 1 h to establish a baseline. Myometrial strips that did not exhibit spontaneous contractions within 2 h after mounting in the device were excluded from further analysis.

Oxytocin stock solutions were prepared at 10 mM in PSS and a dose–response study was initiated by treating tissues with escalating doses of oxytocin (10^–10^ to 10^–8^ M). Recordings of oxytocin-induced tension were made at each concentration at 15-min intervals. At the conclusion of the oxytocin dose–response protocol, the reservoir was replaced with pre-heated high K^+^ solution to confirm contractile integrity.

### Contractility Analysis

Contractility data was recorded with a custom-made program in LabView. Data analysis was conducted using Microsoft Excel and GraphPad Prism (version 9.4.0). We recorded contraction frequency defined as the number of contractions within 15 min. Four additional kinetic parameters were assessed: time to peak tension (*TTP*, s), time to achieve half maximal tension (s), total duration of contraction (*Total Duration of Contractions*, s), and force per cross-sectional area (mN/mm^2^). Baseline contractility data prior to exposure to oxytocin was compared between the normal-weight and obese groups using the Student’s t test. Oxytocin dose–response curves were then created for each contraction parameter using data collected at baseline and with each increasing oxytocin concentration. Two-way ANOVA with Šidák’s multiple comparison test was used to compare the dose response curves in the two groups. A two-tailed p-value < 0.05 was considered significant.

### RNA Extraction and qRT-PCR

Frozen myometrial specimens were thawed, dissected, and placed into RNase-free 1.5- ml microfuge tubes containing 1 ml of TRIzol™ reagent (ThermoFisher) and high-impact glass 1.5 mm beads. Samples were homogenized with a single 180-s round of tissue disruption at 50 Hz using a BeadBug™ 3 microfuge tissue homogenizer (Southern Labware, Cumming, GA). Following addition of chloroform and centrifugation, the aqueous phase was removed and added to an equal volume of 100% ethanol. The mixture was applied to a miRNeasy spin column (Qiagen, Valencia, CA, United States) and processed according to the manufacturer’s protocol. RNA concentrations were quantified using a Nanodrop 2000 spectrophotometer (ThermoFisher, Hudson, NH, United States). Complementary DNA (cDNA) was prepared using oligo(dT)_12–18_ primers with SuperScript III Reverse Transcriptase (Life Technologies, Grand Island, NY, United States). Quantitative PCR was performed using a LightCycler 480 II System (Roche Applied Science, Indianapolis, IN, United States) and the following TaqMan primer/probe sets (Applied Biosystems, Foster City, CA, United States): OXTR, PGFR, and glyceraldehyde 3-phosphate dehydrogenase (GAPDH), a housekeeping gene used for normalization of samples. Assays were run in duplicate and the relative amount of mRNA in each sample was calculated as cycle threshold (CT) values for each gene and normalized to CT values obtained from the housekeeping gene, GAPDH, using the 2^−∆∆CT^ method.

### Protein Extraction and Western Blot Analysis

Frozen myometrial specimens were thawed and homogenized in the BeadBug™ 3 tissue disrupter described above in 1 ml of RIPA buffer (25 mM tris (hydroxymethyl) aminomethane hydrochloride, Tris–HCl, pH 7.6, 150 mM NaCl, 1% NP-40, 1% sodium deoxycholate, DOC, 0.1% sodium dodecylsulfate, SDS) containing protease inhibitor cocktail and 0.1 mM phenylmethylsulfonyl fluoride (PMSF). Total protein was determined using the Bradford assay [25] (Biorad protein assay reagent) using BSA as standard. Samples (40 μg/lane) were fractionated by SDS-PAGE on 4–20% sodium dodecylsulfate-polyacrylamide (SDS-PAGE) NuPAGE™ gels (Invitrogen) and transferred to nitrocellulose membranes using the iBlot™ semi-dry transfer apparatus and iBlot™ Transfer Stacks (Invitrogen). After blocking overnight in 5% non-fat dry milk/Tris-buffered saline with 0.2% Tween-20 (TBST), the blots were probed with antibodies directed against: oxytocin receptor, OXTR; prostaglandin F_2α_ receptor, FP; and glyceraldehyde 3-phosphate dehydrogenase, GAPDH, a housekeeping gene used for normalization of samples. Blots were developed with Clarity™ ECL western blot chemiluminescence reagent (Biorad) and immunoreactive proteins visualized using the Chemidoc™ MP Gel Imaging System (Biorad).

### Receptor Expression Statistical Analysis

Data regarding OXTR and FP gene and protein expression within the myometrial samples are presented as median values with interquartile ranges [IQR]. Grubbs’ test was used to detect outliers and significant outliers were excluded from final analysis. Differences between the normal-weight and obese groups were evaluated using the Mann Whiney U test. The analysis was performed using Prism 10 (GraphPad Software Inc, La Jolla, California).

### Sample Size Calculation

Our preliminary data found that the mean concentration with standard deviation of OXTR gene expression normalized to the housekeeping gene in myometrial tissue from normal-weight patients was 13.2 ± 7.0. Using a two-sided test and assuming alpha of 0.05, we would need to enroll 70 pregnant individuals in a 2:1 ratio of obese to normal-weight participants to detect a 33% difference expression of OXTR receptor with 85% power.

## Results

Seventy-three pregnant individuals, presenting for scheduled cesarean delivery at ≥ 37 weeks’ gestation, were enrolled in the study. Of these participants, 49 (67.1%) had a pre-pregnancy BMI ≥ 30 kg/m^2^, while 24 participants (32.4%) had a pre-pregnancy BMI < 25.0 kg/m^2^. One myometrial tissue sample from the BMI < 25 kg/m^2^ had insufficient quantity to perform receptor expression experiments and was used for the contractility study only. Contractility experiments were only performed in a subset of the total sample. Reasons for this included failure of the myometrial tissue to spontaneously contract, inadequate quantity of the myometrial sample, and/or inability to perform the contractility studies within an appropriate time from sample collection. In total, contractility studies were performed using myometrial samples from 16 participants in the obese group and 9 from the normal-weight group.

The median pre-pregnancy BMI in the obese group was 37.9 kg/m^2^ (interquartile range (IQR) 32.3, 43.0 kg/m^2^), while the median pre-pregnancy BMI was 22.1 kg/m^2^ (IQR 20.6, 23.6 kg/m^2^) among those in the normal-weight group. Maternal demographics were similar between the groups with regards to maternal age, race/ethnicity, and gestational age at delivery (Table 1). Most participants in the study were Non-Hispanic White (69.9%), had at least one prior pregnancy (90.4%), and the most common indication for cesarean was previous cesarean delivery (83.6%).

**Table 1:** Characteristics of participants in pre-pregnancy obesity (BMI ≥ 30 kg/m^2^) and normal-weight (BMI < 25 kg/m^2^) groups

Characteristic	BMI ≥ 30 kg/m^2^ (*n* = 49)	BMI < 25 kg/m^2^ (*n* = 24)
Maternal age (years)	33.4 ± 5.1	31.9 ± 4.9
Parity		
0 1 ≥ 2	3 (6.1) 30 (61.2) 16 (32.7)	4 (16.7) 12 (50.0) 8 (33.3)
Race/ethnicity		
Hispanic Non-Hispanic Black Non-Hispanic White None of the above	2 (4.2) 10 (20.8) 34 (70.8) 2 (4.2)	1 (4.4) 3 (13.0) 17 (73.9) 2 (8.7)
Gestational age	39.0 (38.0, 39.3)	39.1 (39.0, 39.4)
Weight gain during pregnancy (kg)^a^	10.9 (6.3, 15.9)	15.2 (13.6, 19.0)
Delivery BMI (kg/m^2^)^a^	41.1 (36.9, 49.1)	27.5 (25.9, 29.2)
Indication for cesarean		
Prior cesarean Malpresentation Other^b^	42 (85.7) 3 (6.1) 4 (8.2)	19 (79.2) 4 (16.7) 1 (4.17)
Infant birthweight (grams)	3470 (3120, 3910)	3150 (2890, 4340)

Data presented as *n* (%), mean ± standard deviation, or median (interquartile range)

^a^*p*-valve < 0.01

^b^Other indications include prior myomectomy, HSV outbreak, primary elective cesarean delivery

### Contractility Studies

**Fig. 1:**
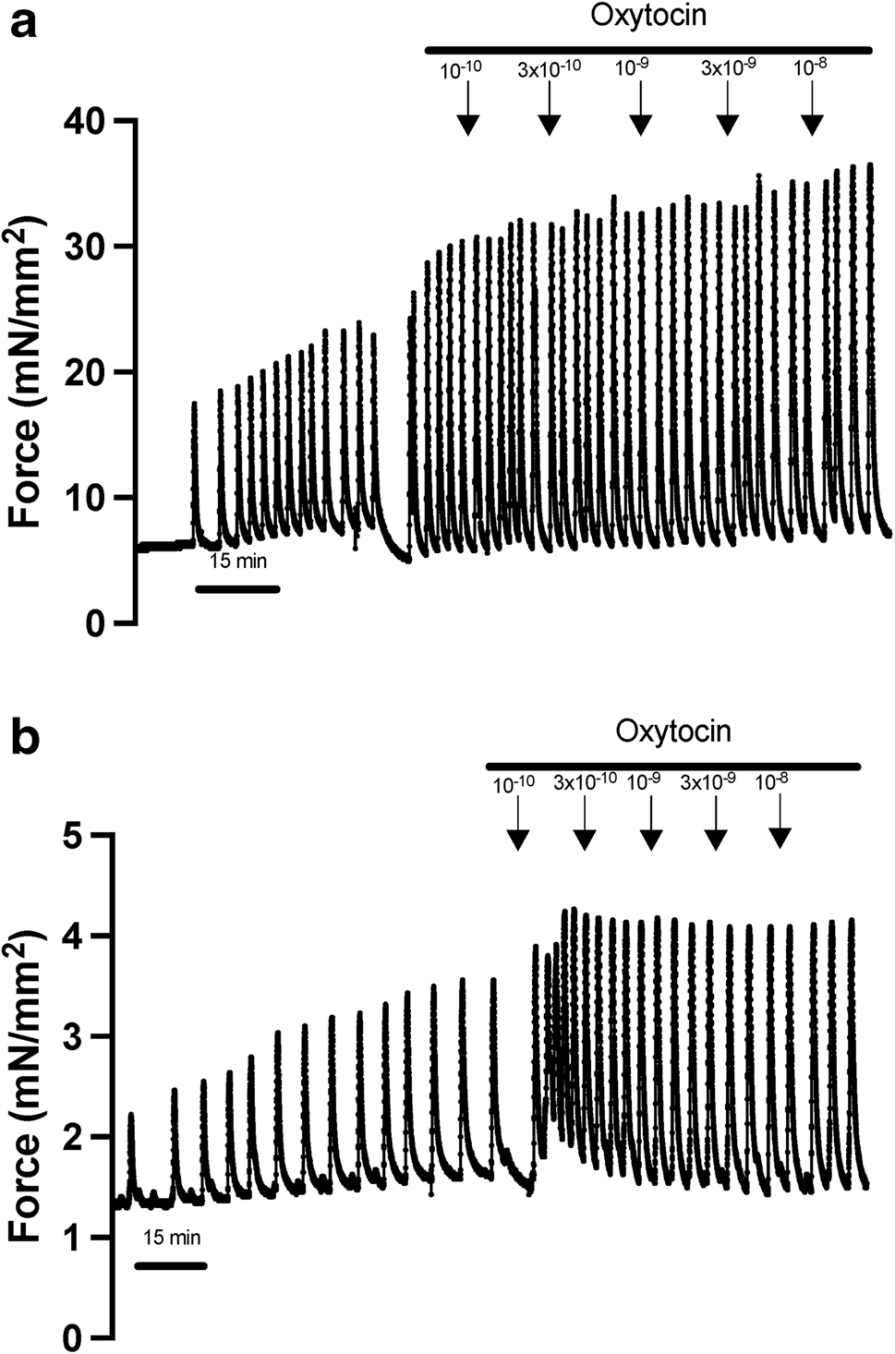
Representative tracing of spontaneous and oxytocin-induced contractions from participants with (**a**) normal BMI (24.6 kg/m^2^) and (**b**) obese BMI (51.7 kg/m^2^). Samples spontaneously contracted and oxytocin was serially added in 15-min increments

**Fig. 2:**
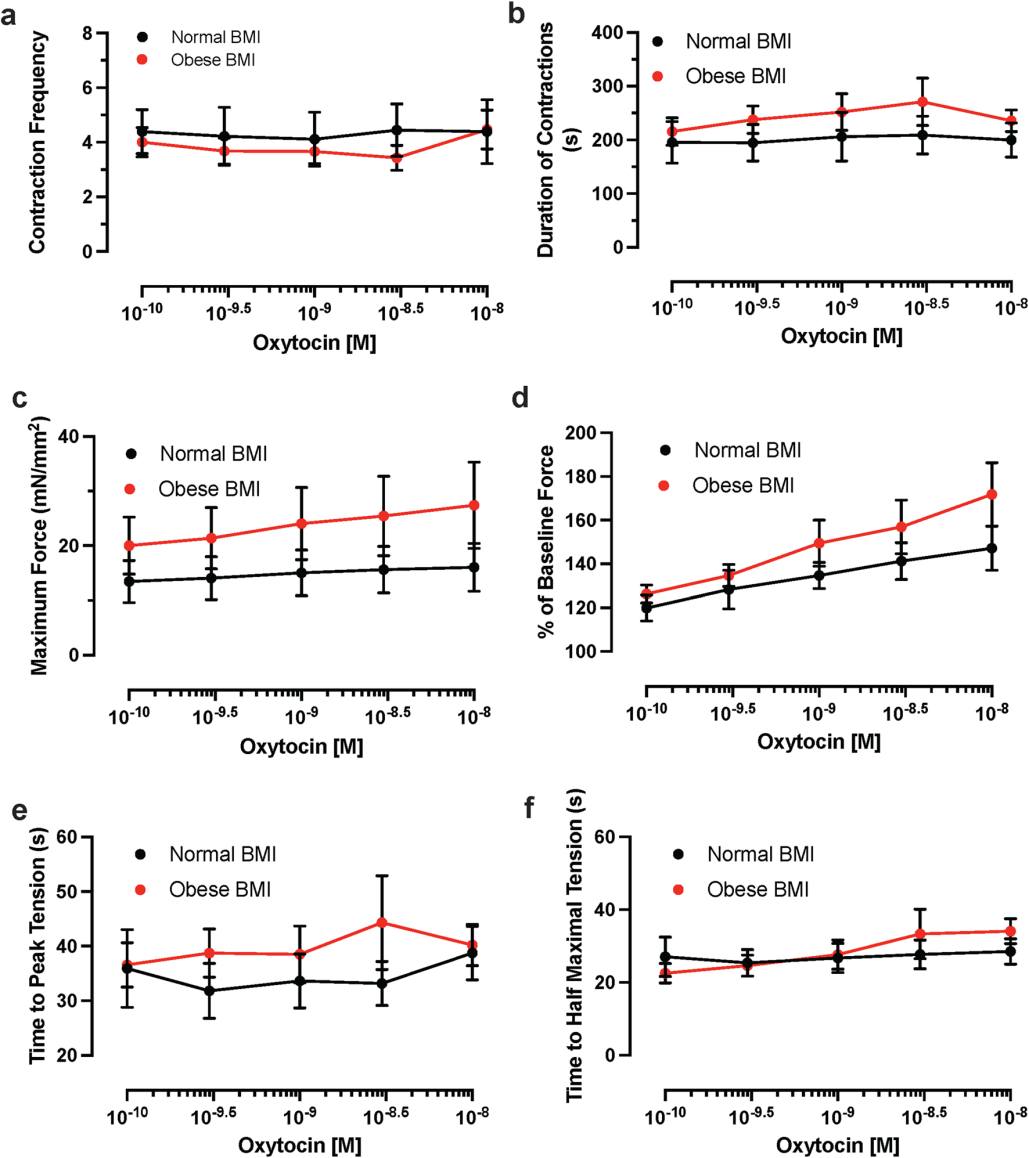
a-e. Comparison of contraction parameters with increasing concentrations of oxytocin in myometrium from normal-weight (BMI < 25 kg/m^2^) and obese (BMI ≥ 30 kg/m^2^) participants. **a** Contraction Frequency, *p* > 0.05; normal-weight *n* = 9, obese *n* = 15 at 10^–10^ and 10^–9^ M, *n* = 14 at 10^–9.5^ and 10^–8.5^ M, *n* = 16 at 10^–8^ M oxytocin. **b** Average duration of contractions (s), *p* > 0.05; normal-weight *n* = 9, obese *n* = 15 at 10^–10^ and 10^–9^ M, *n* = 14 at 10^–9.5^ and 10^–8.5^ M, *n* = 13 at 10^–8^ M oxytocin. **c** Average maximal force of contractions (mN/mm^2^, *p* < 0.05 for normal-weight samples at 10^–10^-10^–8^ M oxytocin compared to baseline value of normal-weight sample per 2-way ANOVA with Šídák’s multiple comparison test; normal-weight *n* = 9, obese *n* = 9 at 10^–10^ and 10^–9^ M, *n* = 8 at 10^–9.5^ and 10^–8^ M, *n* = 7 at 10^–8.5^ M oxytocin. **d** Percentage of baseline force, contractions (mN/mm^2^, *p* < 0.05 for normal-weight samples at 10^–10^-10^–8^ M oxytocin compared to baseline value of normal-weight sample per 2-way ANOVA with Šídák’s multiple comparison test; normal-weight *n* = 9, obese *n* = 9 at 10^–10^ and 10^–9^ M, *n* = 8 at 10^–9.5^ and 10^–8^ M, *n* = 7 at 10^–8.5^ M oxytocin. **e** Average time-to-peak tension (s), *p* > 0.05; normal-weight *n* = 9, obese *n* = 13 at 10^–10^ M and 10^–9^ M, *n* = 12 at 10^–9.5^ and 10^–8^ M, *n* = 11 at 10^–8^ M oxytocin. **f** Average time-to-half-maximal tension (s), *p* > 0.05; normal-weight *n* = 9, obese *n* = 13 at 10^–10^ M and 10^–9^ M, *n* = 12 at 10^–9.5^ and 10^–8^ M, *n* = 11 at 10^–8.5^. Normal-weight and obese samples at each dose of oxytocin were compared to themselves at baseline as well as to the opposing group. Doses administered in 15-min increments. Data presented as average values at each dose with SEM error bars

Baseline spontaneous contraction parameters were similar between the groups (Table 2). Our oxytocin dose–response experiments yielded similar results (Fig. 1). No differences in contraction frequency, duration of contractions, or maximum force were observed between groups (Fig. 2, a-c). There were also no significant differences in force as a percentage of baseline force, time-to-peak tension (TTP) and time to half maximal tension between groups (Fig. 2, d-f). Less than 20% of the samples did not spontaneously contract and were excluded from the contractility study. There were four samples from the obese group that are missing maximum force data as these samples contracted beyond the parameters of the force transducer and settings of the amplifier (KG7, World Precision Instruments) used in the contractility assays, and hence maxed out the force transducer reading on the software.

**Table 2:** Comparison of baseline contraction parameters based on pre-pregnancy BMI

Parameter	BMI ≥ 30 kg/m^2^ (*n* = 16)	BMI < 25 kg/m^2^ (*n* = 9)	*p*
Contraction frequency (n/15 min)	4.5 ± 0.6	4.3 ± 0.8	0.86
Time to peak tension (TTP, s)	31.1 ± 4.5	26.1 ± 4.5	0.46
Time to half maximal tension (s)	22.0 ± 3.1	21.1 ± 2.6	0.84
Total duration (s)	174.9 ± 26.5	165.9 ± 35.4	0.84
Maximum force (mN/mm^2^)	16.3 ± 4.4	11.7 ± 3.5	0.42

Data presented as mean ± standard error of the mean

TTP, time to half maximal tension, total duration: BMI ≥ 30 kg/m^2^, *n* = 12 and BMI < 30 kg/m^2^, *n* = 8. Maximal force: BMI > 30 kg/m^2^, n = 8 and BMI < 25 kg/m^2^, *n* = 8

#### Expression of Oxytocin and Prostaglandin F_2α_ Receptors in Myometrium in Normal-Weight and Obese Groups

**Fig. 3:**
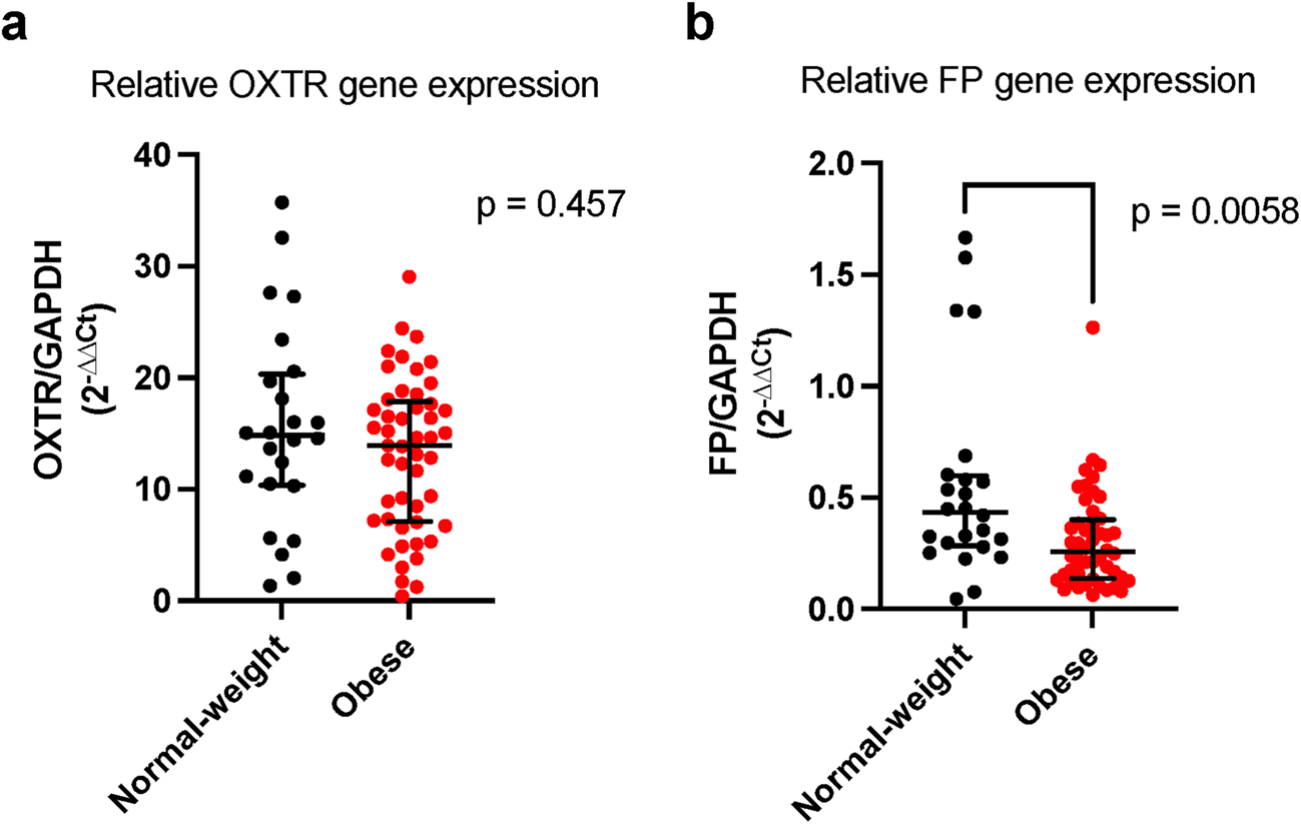
Myometrial OXTR (**a**) and FP gene (**b**) expression in normal-weight and obese pregnant individuals at term. Cycle threshold (CT) values were calculated for each gene and normalized to CT values obtained from the housekeeping gene, GAPDH, using the 2^−∆∆CT^ method. Data are reported as median [IQR].OXTR: *n* = 24 normal-weight, *n* = 49 obese. FP: *n* = 24 normal-weight, *n* = 48 obese

**Fig. 4:**
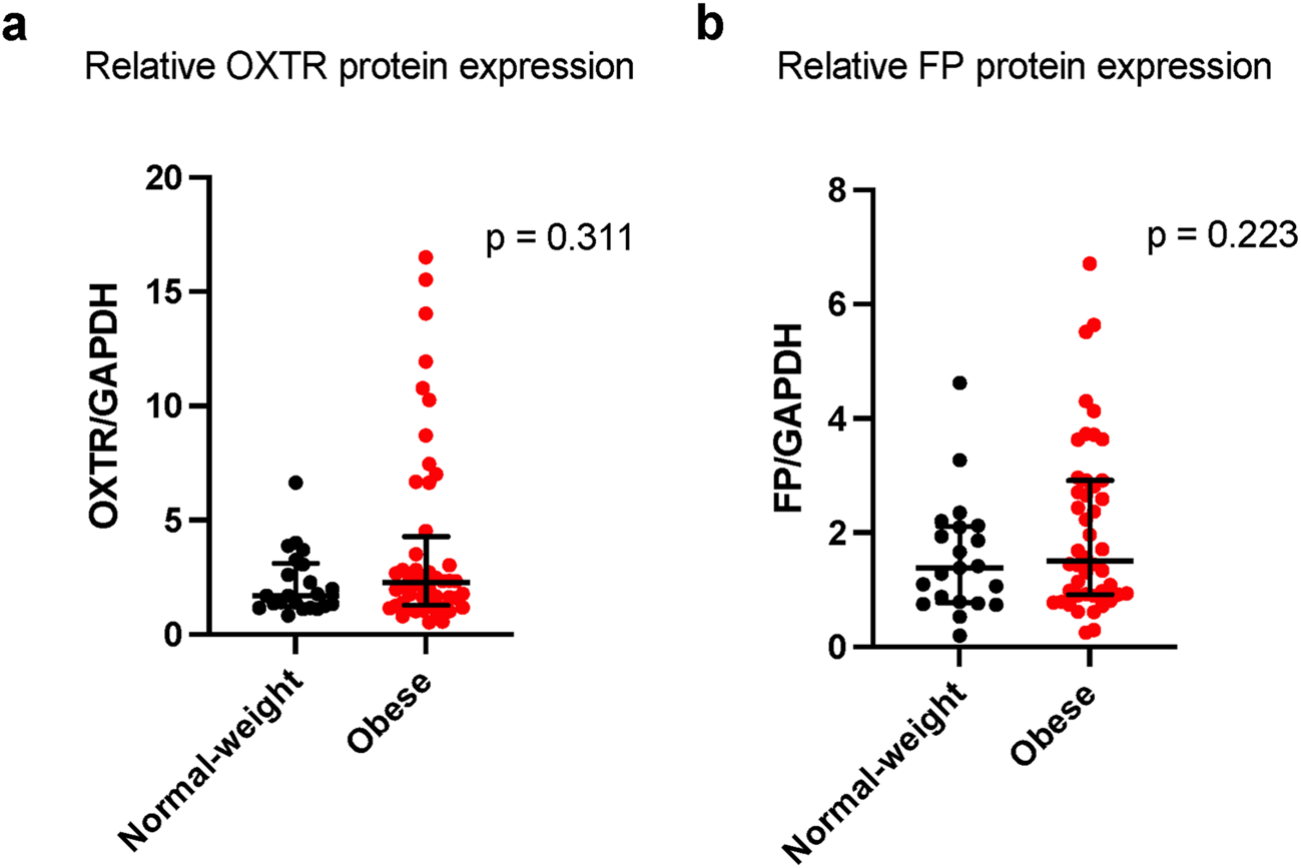
Myometrial OXTR (**a**) and FP (**b**) protein expression in normal-weight and obese pregnant individuals at term. Data are reported as median [IQR]. OXTR: *n* = 22 normal-weight, *n* = 48 obese. PGFR: *n* = 21 normal-weight, *n* = 46 obese

**Fig. 5:**
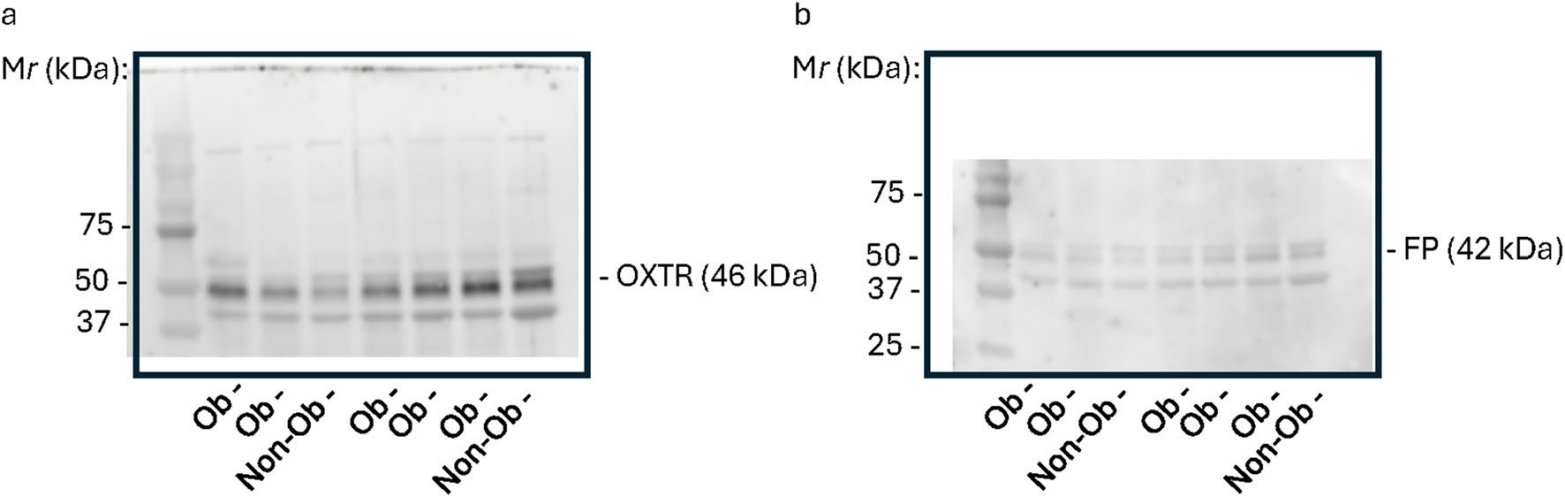
Representative western blots. **a** Myometrial samples probed with antibodies directed against oxytocin receptor (OXTR). **b** Myometrial samples probed with antibodies against prostaglandin receptor (FP). “Ob” represents a sample from an individual with obesity and “Non-Ob” represents a sample from a normal-weight individual

Analysis of mRNA transcripts indicated that the myometrial OXTR gene expression did not differ between normal-weight and obese pregnant individuals (*p* = 0.46), whereas myometrial FP gene expression was significantly lower in the obese group compared to those with normal BMI (*p* < 0.01) (Fig. 3). Protein expression of the OXTR and FP, however, did not differ between the groups (Fig. 4). Representative western blots are shown in Fig. 5.

## Discussion

In our prospective cohort study, we found that spontaneous and oxytocin-induced contractility was similar in term myometrial samples from pregnant individuals with obesity compared to those with normal BMI. Additionally, there were no differences in expression of either oxytocin or prostaglandin F_2α_ receptors, which are key components within the signaling pathways leading to myometrial contractions in pregnancy.

The role of maternal obesity in dysfunctional labor and increased risk of cesarean delivery is a prominent issue in obstetrics, but the mechanisms that underpin the clinical observation are not understood [8, 16, 26]. One way to address the issue of myometrial function in the context of obesity is to measure uterine muscle contractility using a well-established *ex vivo* model [23]. In this system, physiological parameters (e.g., contraction frequency, duration and force) can be measured using small segments of myometrium isolated from pregnant individuals of various pre-pregnancy BMI values to ascertain whether maternal adiposity influences contractility. The main findings of our study indicate that myometrial sample contractility, including contraction frequency, duration and generated force, do not differ significantly between obese and normal-weight individuals, using pre-pregnancy BMI as the measure of adiposity. These results are consistent with those reported by Higgins et al. in a larger cohort of individuals, in which they found no significant association between maternal BMI and measures of contractility in myometrial samples obtained at time of cesarean [19]. Other investigators have observed differences in myometrial contractility in obese and non-obese individuals using similar laboratory techniques, but their results have been inconsistent. For example, one group found higher BMI was associated with lower contraction force and frequency [16], while another group found that higher maternal BMI was associated with increased myometrial contractile force but no difference in frequency [17]. We also expanded upon the work of other researchers by evaluating additional muscle kinetic parameters including time to peak tension and time to half-maximal tension, albeit no differences were observed.

To better understand the potential biomolecular mechanisms by which obesity could affect myometrial function, we investigated myometrial gene and protein expression of OXTR and FP receptor. Our findings suggest that maternal BMI is not associated with differential myometrial expression of OXTR. This supports findings from Grotegut et al. whose work also demonstrated no association between maternal BMI and OXTR expression [22]. Activation of OXTR in myometrial cells and other gestational tissues induces a signaling cascade that ultimately leads to the generation of myometrial contractions. One of the downstream effects of oxytocin binding to its receptor is the formation of prostaglandins. Prostaglandins, such as PGF_2α_, bind to prostaglandin receptor, FP, on myometrial cells to promote myometrial contractility [20]. In our study, myometrial samples from pregnant individuals with obesity at term had significantly reduced FP gene expression compared to myometrial samples from normal-weight individuals, yet we detected no difference in the protein expression of the FP. Further research is needed to better define this possible association. To our knowledge, this is the first study to investigate the association between maternal BMI and FP expression in the myometrium in pregnancy.

The present investigation has both strengths and limitations. First, we recruited only non-laboring individuals avoiding previous myometrial activation *in vivo* and included only samples that exhibited spontaneous and K^+^-activated contractile activity once placed in the *ex vivo* system. Second, we attempted to correlate contractile activity with the expression of two major uterotonic receptor systems, OXTR [20] and FP [27]. Another strength was the high proportion of participants with obesity in our study. Similar studies that have assessed the association between maternal BMI and uterine contractility have included a relatively small proportion of patients with obesity. For example, Higgins et al. performed a study with samples from 85 pregnant individuals but only 22 (25.9%) had a BMI ≥ 30 kg/m^2^ and only 3 had class III obesity (BMI ≥ 40 kg/m^2^) [19], whereas the median BMI in our obese group was 37.9 kg/m^2^.

A limitation of our study is the small sample size, particularly with regards to the performance of contractility experiments, which were only performed in a third of the total samples due to availability of research team member with the expertise to perform the studies in the appropriate timeframe as well as adequacy of the samples. Moreover, there was considerable variability in both contractile activity and expression of relevant receptors (OXTR and FP) that are the initiators of uterotonic signaling in the specimens. The fact that only small segments of myometrium from a single region of the uterus are used for the *ex vivo* contractility measurements make it a formidable challenge to ascertain a thorough picture of the role of obesity in uterine activity. Samples were also collected from individuals undergoing pre-labor cesarean delivery, thus may not adequately reflect differences in myometrial function during labor. Ideally, measuring uterine activity *in vivo* would be preferable, but this is technically challenging in humans.

The decision to use pre-pregnancy BMI rather than BMI at time of delivery is also controversial but is consistent with approaches used by other investigators who have evaluated the influence of BMI on *in vitro* myometrial contractility studies in pregnancy [16, 18, 19]. A leading theory related to obesity and labor dysfunction is that metabolic and hormonal factors related to adiposity influence myometrial contractility [28]. As it is possible that individuals with normal pre-pregnancy BMI (18.5–24.9 kg/m2) can have a BMI categorized as overweight or even obese with recommended weight gain, pre-pregnancy BMI may better identify individuals with metabolic and hormonal phenotypes characteristic of obesity than delivery BMI. An evaluation of the interaction between pre-pregnancy BMI and gestational weight gain on myometrial function would of great interest in the future but would require a larger study.

## Conclusion

Our study demonstrated no association between maternal obesity and intrinsic myometrial contractility. These results suggest alternative mechanisms for the higher rates of labor dysfunction observed clinically in individuals with obesity. Additionally, our results show that differences in oxytocin receptor expression are also unlikely to explain these clinical observations. More research to expand our understanding of the mechanisms underlying the relationship between maternal BMI and labor progress is clearly needed.

## Data Availability

The data that support the findings of this study are available from the corresponding author upon reasonable request. However, access is restricted by the limitations in the ethical approval (including patient informed consent).

## References

[1] Flegal KM, Kruszon-Moran D, Carroll MD, Fryar CD, Ogden CL. Trends in obesity among adults in the United States, 2005 to 2014. JAMA. 2016;315(21):2284–91. 10.1001/jama.2016.6458. 27272580 10.1001/jama.2016.6458 PMC11197437

[2] Walsh SW. Obesity: a risk factor for preeclampsia. Trends Endocrinol Metab. 2007;18(10):365–70. 10.1016/j.tem.2007.09.003. 18023357 10.1016/j.tem.2007.09.003

[3] Bicocca MJ, Mendez-Figueroa H, Chauhan SP, Sibai BM. Maternal obesity and the risk of early-onset and late-onset hypertensive disorders of pregnancy. Obstet Gynecol. 2020;136(1):118–27. 10.1097/AOG.0000000000003901. 32541276 10.1097/AOG.0000000000003901

[4] Chu SY, Callaghan WM, Kim SY, Schmid CH, Lau J, England LJ, et al. Maternal obesity and risk of gestational diabetes mellitus. Diabetes Care. 2007;30(8):2070–6. 10.2337/dc06-2559a. 17416786 10.2337/dc06-2559a

[5] D’Souza R, Horyn I, Pavalagantharajah S, Zaffar N, Jacob CE. Maternal body mass index and pregnancy outcomes: a systematic review and metaanalysis. Am J Obstet Gynecol MFM. 2019;1(4):100041. 10.1016/j.ajogmf.2019.100041. 33345836 10.1016/j.ajogmf.2019.100041

[6] Dai RX, He XJ, Hu CL. Maternal pre-pregnancy obesity and the risk of macrosomia: a meta-analysis. Arch Gynecol Obstet. 2018;297(1):139–45. 10.1007/s00404-017-4573-8. 29080962 10.1007/s00404-017-4573-8

[7] Polnaszek BE, Raghuraman N, Lopez JD, Frolova AL, Wesevich V, Tuuli MG, et al. Neonatal morbidity in the offspring of obese women without hypertension or diabetes. Obstet Gynecol. 2018;132(4):835–41. 10.1097/AOG.0000000000002775. 30130347 10.1097/AOG.0000000000002775 PMC7202404

[8] Chu SY, Kim SY, Schmid CH, Dietz PM, Callaghan WM, Lau J, et al. Maternal obesity and risk of cesarean delivery: a meta-analysis. Obes Rev. 2007;8(5):385–94. 10.1111/j.1467-789X.2007.00397.x. 17716296 10.1111/j.1467-789X.2007.00397.x

[9] Kawakita T, Reddy UM, Landy HJ, Iqbal SN, Huang CC, Grantz KL. Indications for primary cesarean delivery relative to body mass index. Am J Obstet Gynecol. 2016;215(4):515 e1–9. 10.1016/j.ajog.2016.05.023. 10.1016/j.ajog.2016.05.023 PMC5045770 27210064

[10] Kominiarek MA, Vanveldhuisen P, Hibbard J, Landy H, Haberman S, Learman L, et al. The maternal body mass index: a strong association with delivery route. Am J Obstet Gynecol. 2010;203(3):264 e1–7. 10.1016/j.ajog.2010.06.024. 10.1016/j.ajog.2010.06.024 PMC2933947 20673867

[11] Kominiarek MA, Zhang J, Vanveldhuisen P, Troendle J, Beaver J, Hibbard JU. Contemporary labor patterns: the impact of maternal body mass index. Am J Obstet Gynecol. 2011;205(3):244 e1–8. 10.1016/j.ajog.2011.06.014. 10.1016/j.ajog.2011.06.014 PMC3212654 21798510

[12] Norman SM, Tuuli MG, Odibo AO, Caughey AB, Roehl KA, Cahill AG. The effects of obesity on the first stage of labor. Obstet Gynecol. 2012;120(1):130–5. 10.1097/AOG.0b013e318259589c. 22914401 10.1097/AOG.0b013e318259589c PMC4494673

[13] Blomberg M. Maternal obesity and risk of postpartum hemorrhage. Obstet Gynecol. 2011;118(3):561–8. 10.1097/AOG.0b013e31822a6c59. 21860284 10.1097/AOG.0b013e31822a6c59

[14] Conner SN, Verticchio JC, Tuuli MG, Odibo AO, Macones GA, Cahill AG. Maternal obesity and risk of postcesarean wound complications. Am J Perinatol. 2014;31(4):299–304. 10.1055/s-0033-1348402. 23765707 10.1055/s-0033-1348402 PMC3796045

[15] Krieger Y, Walfisch A, Sheiner E. Surgical site infection following cesarean deliveries: trends and risk factors. J Matern Fetal Neonatal Med. 2017;30(1):8–12. 10.3109/14767058.2016.1163540. 27023698 10.3109/14767058.2016.1163540

[16] Zhang J, Bricker L, Wray S, Quenby S. Poor uterine contractility in obese women. BJOG. 2007;114(3):343–8. 10.1111/j.1471-0528.2006.01233.x. 17261121 10.1111/j.1471-0528.2006.01233.x

[17] Crankshaw DJ, O’Brien YM, Crosby DA, Morrison JJ. Maternal body mass index and spontaneous contractility of human myometrium in pregnancy. J Perinatol. 2017;37(5):492–7. 10.1038/jp.2016.271. 28125101 10.1038/jp.2016.271

[18] Luca AM, Carvalho JCA, Ramachandran N, Balki M. The effect of morbid obesity or advanced maternal age on oxytocin-induced myometrial contractions: an in vitro study. Can J Anaesth. 2020;67(7):836–46. 10.1007/s12630-020-01615-6. 32189217 10.1007/s12630-020-01615-6

[19] Higgins CA, Martin W, Anderson L, Blanks AM, Norman JE, McConnachie A, et al. Maternal obesity and its relationship with spontaneous and oxytocin-induced contractility of human myometrium in vitro. Reprod Sci. 2010;17(2):177–85. 10.1177/1933719109349780. 19828431 10.1177/1933719109349780

[20] Arrowsmith S, Wray S. Oxytocin: its mechanism of action and receptor signalling in the myometrium. J Neuroendocrinol. 2014;26(6):356–69. 10.1111/jne.12154. 24888645 10.1111/jne.12154

[21] Garabedian MJ, Hansen WF, McCord LA, Manning MA, O’Brien JM, Curry TE Jr. Up-regulation of oxytocin receptor expression at term is related to maternal body mass index. Am J Perinatol. 2013;30(6):491–7. 10.1055/s-0032-1329179. 23355275 10.1055/s-0032-1329179

[22] Grotegut CA, Gunatilake RP, Feng L, Heine RP, Murtha AP. The influence of maternal body mass index on myometrial oxytocin receptor expression in pregnancy. Reprod Sci. 2013;20(12):1471–7. 10.1177/1933719113488446. 23653389 10.1177/1933719113488446 PMC3817668

[23] Arrowsmith S, Keov P, Muttenthaler M, Gruber CW. Contractility Measurements of Human Uterine Smooth Muscle to Aid Drug Development. J Vis Exp. 2018;131. 10.3791/56639. 10.3791/56639 PMC5841565 29443077

[24] Milani-Nejad N, Chung JH, Canan BD, Davis JP, Fedorov VV, Higgins RSD, et al. Insights into length-dependent regulation of cardiac cross-bridge cycling kinetics in human myocardium. Arch Biochem Biophys. 2016;601:48–55. 10.1016/j.abb.2016.02.005. 26854725 10.1016/j.abb.2016.02.005 PMC4899103

[25] Bradford MM. Rapid and sensitive method for the quantitation of microgram quantities of protein utilizing the principle of protein-dye binding. Anal Biochem. 1976;72(1–2):248–54. 942051 10.1016/0003-2697(76)90527-3

[26] Arrowsmith S, Wray S, Quenby S. Maternal obesity and labour complications following induction of labour in prolonged pregnancy. BJOG. 2011;118(5):578–88. 10.1111/j.1471-0528.2010.02889.x. 21265999 10.1111/j.1471-0528.2010.02889.x PMC3085126

[27] Grigsby PL, Sooranna SR, Adu-Amankwa B, Pitzer B, Brockman DE, Johnson MR, et al. Regional expression of prostaglandin E2 and F2alpha receptors in human myometrium, amnion, and choriodecidua with advancing gestation and labor. Biol Reprod. 2006;75(2):297–305. 10.1095/biolreprod.106.051987. 16707767 10.1095/biolreprod.106.051987

[28] Azais H, Leroy A, Ghesquiere L, Deruelle P, Hanssens S. Effects of adipokines and obesity on uterine contractility. Cytokine Growth Factor Rev. 2017;34:59–66. 10.1016/j.cytogfr.2017.01.001. 28389056 10.1016/j.cytogfr.2017.01.001

